# Gastroparesis with bezoar formation in patients treated with glucagon-like peptide-1 receptor agonists: potential relevance for bariatric and other gastric surgery

**DOI:** 10.1093/bjsopen/zrac169

**Published:** 2023-02-01

**Authors:** Veronica Preda, Skylar Su-Yee Khoo, Tamara Preda, Reginald V Lord

**Affiliations:** Department of Endocrinology, Human and Health Sciences Macquarie University, Sydney, Australia; Department of Endocrinology, Human and Health Sciences Macquarie University, Sydney, Australia; Department of Surgery, University of Notre Dame School of Medicine, Sydney, Australia; Department of Surgery, University of Notre Dame School of Medicine, Sydney, Australia

*Dear Editor*

Glucagon-like peptide-1 receptor agonist (GLP-1RA) medications are frequently prescribed for the treatment of type 2 diabetes mellitus (T2DM) and for weight loss in overweight or obese patients with or without T2DM^1^. Following an oral glucose load, GLP-1RAs bind to the corresponding GLP-1 receptor in pancreatic β cells to stimulate glucose-dependent insulin release while reducing glucagon secretion from pancreatic α cells, with a resultant fall in serum glucose concentration. GLP-1RA drugs are effective in lowering HbA1c whilst having a low risk of hypoglycaemia and may provide cardiorenal benefits in patients with T2DM^1^. The importance of GLP-1-mediated effects was noted by Casella *et al.*^2^, who reported a positive correlation between insulin sensitivity, loss of excess bodyweight, and GLP-1 levels in a sleeve gastrectomy cohort followed for 12 months.

Initially these medications required weekly injection; however, a GLP-1RA tablet (oral semaglutide, Rybelsus) has been released, which is expected to greatly increase numbers of patients treated with GLP-1RA therapy. GLP-1RA side effects include nausea, vomiting, and diarrhoea. A case of bezoar formation associated with GLP-1RA use has been reported^3^. The aims of our study were to provide preliminary estimates of the frequency of bezoar formation in patients taking GLP-1RA medications and the time to resolution of bezoars after ceasing these medications.

Data were collected prospectively and retrospectively in a series of 100 consecutive fasting patients who underwent routine gastroscopy before sleeve gastrectomy, regardless of the presence or absence of symptoms. Institutional low-risk ethics approval without the need for written individual patient consent (St Vincent’s Hospital ETH10886) was obtained.

Basic demographics of the patients are shown in *Table 1*. Four of 23 (17.4 per cent) patients who were being treated with GLP-1RA medications were found to have moderately large gastric bezoars at gastroscopy (diameter 4 cm or more), whereas 0 of the 77 patients who were not receiving these medications had bezoars. Two patients with bezoars had diabetes, one had prediabetes, and one did not have diabetes. Three of the patients with bezoars had nausea, which resolved promptly after ceasing GLP-1RA therapy. One patient had a nuclear medicine scan showing abnormal gastric emptying (76 per cent retention of a solid meal at 4 h (normal less than 10 per cent retention)) whilst taking liraglutide. A repeat nuclear medicine scan at 2 weeks after ceasing liraglutide was normal. Normal emptying was also demonstrated by nuclear medicine scanning 1 week after ceasing semaglutide in one of the patients with T2DM and 3 weeks after ceasing semaglutide in the patient with prediabetes. Repeat endoscopy demonstrated no bezoars after cessation of GLP-1RA therapy in all four patients.

**Table 1:** Characteristics of 100 consecutive patients

	Patients on GLP-1RA (*n* = 23)	Patients not on GLP-1RA (*n* = 77)
Sex ratio M:F (%)	6:17 (26:74)	23:54 (30:70)
Age (years), median (range)	50 (23–64)	45 (19–73)
BMI (kg/m^2^), median (range)	37 (32–51)	41 (29–66)
T2DM	12 (52.2)	12 (15.6)

Values are *n* (%) unless otherwise stated. GLP-1RA, glucagon-like peptide-1 receptor agonist; T2DM, type 2 diabetes mellitus.

We consider it most likely that these bezoars occurred due to delayed gastric emptying caused by GLP-1RA therapy, as the bezoars were not present after the therapy was ceased and because delayed gastric emptying in some patients is a reported side effect of GLP-1RA drugs^2,4,5^. Larger studies are required for a more accurate assessment of bezoar incidence. Our findings may be significant due to the potential for bezoars to cause intestinal obstruction. We surmise that some degree of obstruction could occur after surgery in patients undergoing gastric or bariatric surgery, in particular sleeve gastrectomy but possibly also bypass surgery. The presence of a bezoar could also cause inadequate stapler firing and staple line leak if the bezoar was included in the stapler. Surgeons may consider performing gastroscopy to check for bezoar formation before performing gastric surgery in patients taking GLP-1RA medications. Relying on symptoms alone to decide the need for gastroscopy may be unreliable. Normal gastric emptying seems to return within a few weeks after ceasing these drugs, with clearance of the bezoar. Finally, surgeons should be aware of GLP-1RA drugs as a cause of nausea and vomiting, as they may be referred patients with these symptoms who only require medication cessation for symptom relief.

## Data Availability

The data set is available at https://researchonline.nd.edu.au/med_article/1471/.
